# Editorial: Socio-gerontechnology—New perspectives on the digital transformation of later life

**DOI:** 10.3389/fsoc.2023.1183572

**Published:** 2023-03-31

**Authors:** Vera Gallistl, Stephen Katz, Franz Kolland, Alexander Peine

**Affiliations:** ^1^Division Gerontology and Health Research, Department General Health Studies, Karl Landsteiner University of Health Sciences, Krems an der Donau, Austria; ^2^Sociology Department, Trent University, Peterborough, ON, Canada; ^3^Department of Cultural Studies, Open University of the Netherlands, Heerlen, Netherlands

**Keywords:** aging, materiality, digital technology, gerontology, critical sociology

Digital infrastructures are increasingly integrated into the everyday lives of older people. They shape the experiences and constitution of age and aging. Consequently, the sociology of age and aging has turned to technology use in later life as a topic of research. Many such gerontological studies on aging and technologies, however, are rather applied and techno-optimist, asking how technology can improve older people's lives (Schulz et al., 2015). Recently, more critical and cultural approaches in the sociology of age and aging and other “critical” fields like critical studies of science and technology and of data have sought to move away from such interventionist forms of theorizing that are often used to make sense of the digital engagements of older adults (Peine and Neven, 2019). These approaches have criticized, on the one hand, ageist stereotypes about technology use in later life in design processes and the paternalist stance toward older adults resulting from it (Neven, 2010; Mannheim et al., 2022) and, on the other, the techno-optimism of gerontological research on digital technologies (Moreira, 2017).

Critical approaches and theories to aging and technology – now widely referred to as Socio-gerontechnology—have zoomed in on the social, infrastructural, cultural and material forces through which aging, care, health and technology already shape and have shaped each other (Peine et al., 2021). This includes, on the one hand, a deeper empirical engagement with design processes and innovation policy to highlight how new technologies not only address alleged problems or challenges of individual or population aging, but how they create and select certain ideas about aging that work well as targets for design or innovation policies (Bischof and Jarke, 2021; Peine and Neven, 2021). In such a view, design processes and innovation policy become important sites for our empirical understanding of age and aging, too, because they produce and reinforce societally shared ideas of how we can and should age—ideas whose contingent nature can be revealed by empirical inquiry (Lipp and Peine, 2022).

On the other hand, Socio-gerontechnology scholars have also deeply engaged with the everyday life of older people, aging bodies and the construction of age and aging in relation to technology. These engagements highlighted the many generative and creative processes through which technologies are shaped and obtain meaning in encounters with older people. In this view, the many “little arrangements” (López Gómez, 2015) that already make up the lives of older people are relevant to understand how digital infrastructures come to matter in the lives of older adults and their families, friends and care givers. It also becomes apparent how age and aging—as a lived experience and socio-material construct—is and has always been shaped in relation to diverse forms of materiality, including technologies (Höppner and Urban, 2018). Critical studies, then, have highlighted how the effects of digital infrastructures cannot be reduced to certain forms of pre-defined “impacts” against which they can be evaluated. Rather, their multiple and emergent forms in the lives of older people become visible (Ertner and Lassen, 2021) as a background against which the relevance of new technologies can be discussed. Others have highlighted how aging is increasingly quantified through smart and wearable technologies, and how this quantification of aging creates and ambiguous image of the aging body as both constantly improvable and inevitably in decline (Katz and Marshall, 2018).

This Research Topic brings together critical approaches that explore and theorize the digital transformation of later life, taking the digital, social and material aspects that make and shape later life into account and drawing upon theories from age studies, critical, cultural and social gerontology, materialist sociology, STS or critical data studies. Its six contributions highlight the ways aging is co-constituted in relation to technologies and problematize different aspects of the relational terrain between aging and technologies. They also stimulate new directions in theorizing and empirically exploring the manifold relations between aging and technology. Three topics emerge as cross-cutting issues:

First, the contributions to this Research Topic expand thinking about digital ageism, in highlighting new fields and mechanisms through which ageism emerges and becomes visible in technology design and implementation. Berridge and Grigorovich use surveillance technologies as a starting point to raise important questions about the connections between ageism and algorithmic technologies. Importantly, they situate ageism as a structural force, and argue that the existing ageism in nursing homes has been exacerbated—rather than newly implemented—through algorithmic technologies. Graham introduces “*ambient ageism”* as a concept to problematize the musical discourse of AgeTech advertisements and, drawing on Van Leeuwen (2012) framework for critical analysis of musical discourse, lays out methodological tools to empirically explore how ageism sounds in relation to different forms of technology.

Second, the authors contributing to this Research Topic invite us to explore and theorize about data and data representations as an important site for the socio-material constitution of age and aging. Ellison et al. examine the datafication of aging by analyzing visualizations of data in promotional images of smart sensor technologies. They find an (in)visibility paradox in the visual representation of older bodies and their data: While the visibility of older bodies is central, its visibility is limited to the data produced through technological devices that are focused on specific aspects of movement or patterns of behavior. Stypińska and Franke address the issue of age bias in artificial intelligence (AI) and argue that the flawed representation of older adults in data is of major concern here. Drawing on scholarship in critical data studies, they argue that the current misrepresentation older adults in big data infrastructures situates them as “*vulnerable data subjects*”, ultimately contributing to a negative imaginary of aging and later life.

Lastly, the contributions to this Research Topic focus our attention to the ways in which the relationship between aging and technologies can be imagined otherwise. Sheahan engages with the design of technologies for older adults, guided by Fischer et al. (2021) question: “What characterizes “better” images of aging created by designers?”. Sheahan proposition is that rather than determining “good” imaginaries of older adults, offering more transparency in the processes of production of these images in technology design is a fruitful pathway into the future. Following Adorno's critical theory, Leontowitsch et al. theorize intergenerational learning arrangements as spaces in which incongruences of post-digital worlds can be reflected, reassessed, contained and ultimately re-imagined with older and younger generations. They also assess digital and social inequalities in later life as part of critical socio-gerontechnology. Ultimately, the contributions to this Research Topic hence not only provide in-depth analyses into several fields of research that are relevant to Socio-Gerontechnology, but also invite us to critically think about how we can re-imagine the relationship between aging and technologies beyond existing practices.

## Author contributions

VG and AP wrote the first version of the manuscript. SK and FK reviewed and edited it. All authors listed have made a substantial, direct, and intellectual contribution to the work and approved it for publication.
